# Predictors of Initial CPAP Prescription and Subsequent Course with CPAP in Patients with Central Sleep Apneas

**DOI:** 10.21203/rs.3.rs-3199807/v1

**Published:** 2023-07-27

**Authors:** Brian W. Locke, Jeffrey Sellman, Jonathan McFarland, Francisco Uribe, Kimberly Workman, Krishna M. Sundar

**Affiliations:** University of Utah; Boston University; Michigan State University; University of Utah; University of Utah

**Keywords:** Sleep apnea, central, Sleep apnea syndromes, Continuous Positive Airway Pressure, Clinical Decision-Making

## Abstract

**Purpose:**

Guidelines recommend considering an initial trial of continuous positive airway pressure (CPAP) to treat central sleep apnea (CSA). However, practice patterns vary widely. This study investigated predictors for an initial trial of CPAP in patients with central apneas and whether those factors predict adequate treatment response in patients receiving an initial CPAP trial.

**Methods:**

Charts of patients receiving a diagnostic code for CSA following a sleep study during 2016–2018 at a single center were reviewed. Patient factors, initial treatment prescriptions, and subsequent changes to therapy were extracted from electronic health records. Regression models were used to estimate factors associated with an initial CPAP prescription and the likelihood of an adequate CPAP response (no subsequent therapy change or nonadherence) among patients prescribed CPAP.

**Results:**

429/588 (73%) patients with central apneas received an initial trial of CPAP. Younger age, diagnosis by home sleep testing, non-opiate etiology of central apneas, and a lower proportion of central apneas at diagnosis were independently associated with a higher likelihood of an initial CPAP trial. A lower proportion of central apneas was associated with a higher probability of adequate response, while current smoking and opiate-related central apneas predicted an unsuccessful CPAP trial. A new finding was that older age predicted a lower likelihood of an initial CPAP prescription but did not predict a suboptimal response to CPAP.

**Conclusion:**

Clinicians may incorrectly weigh certain clinical and sleep study characteristics when deciding whether to trial CPAP for patients with central apneas.

## Introduction

Sleep apneas (complete cessation of airflow) and hypopneas (reduction in airflow associated with oxygen desaturation) are classified as obstructive or central based on whether respiratory muscle effort is generated during the apnea or hypopnea [1]. This dichotomization simplifies the classification of respiratory abnormalities but does not predict which patients will respond to continuous positive airway pressure (CPAP) therapy [2]. The modern taxonomy of central sleep apnea (CSA) recognizes at least two mechanisms leading to central apneas or hypopneas: ventilatory control instability and failure of rhythm generation [3]. Differences in these features may explain why CPAP may be less effective in some patients with CSA, such as those with CSA from opiate use [4].

Considering CPAP for initial therapy is recommended for all common etiologies of CSA, though data on the comparative effectiveness of different approaches is sparse [5; 6]. As initial therapy, CPAP is faster to set up, cheaper, and might be safer in some patients than alternative treatments such as adaptive servo ventilation (ASV) [7; 8]. Alternatively, patients with CSA that fail to resolve with CPAP may have poor adherence due to the persistence of CSA-related symptoms that can potentially lower their willingness to trial or adhere to subsequent positive airway pressure (PAP)-based sleep treatments [9; 10]. Thus, presumably, providers do not prescribe a trial of CPAP to patients they perceive to be at higher risk of having an inadequate treatment response. Yet, little is known about the accuracy of these implied predictions. CPAP usage might be improved if subsets of patients could be identified that are currently unlikely to receive CPAP but favorably respond when it is trialed, or vice versa.

We sought to model the relative importance of several demographic, anthropomorphic, and disease characteristics on sleep clinicians’ decision to trial CPAP in patients with central apneas. We then evaluated whether these factors also predicted an adequate response to CPAP as indicated by no subsequent changes in treatment modality or nonadherence.

## Methods

### Study design:

We performed a retrospective review of electronic medical records of patients with central apneas at the University of Utah Sleep-Wake Center. The University of Utah Sleep Medicine program is an American Academy of Sleep Medicine-accredited sleep laboratory at an altitude of 4500 feet in Salt Lake City, Utah. The study was approved by the University of Utah Institutional Review Board with a waiver of individual informed consent (IRB # 00123537).

### Subjects:

Patients were eligible for inclusion if they were age 18 or older and had received an International Classification of Diseases (ICD)-10 diagnosis codes for CSA (G47.31, G47.32, G47.37, G47.39, R06.3) following an outpatient polysomnogram (PSG) or home sleep apnea test (HSAT) using a Type III portable sleep testing device (Nox T3, Nox Medical USA, Suwanee, GA, USA) between January 2016 to December 2018 (reflecting post-SERVE-HF trial prescribing patterns [7]). Patients were excluded if they had no follow-up after diagnostic testing. Patients with inpatient sleep testing were excluded due to a preliminary review showing low rates of follow-up care within our institution.

### Data collection:

A manual chart review of sleep testing, progress notes of all specialty types, pharmacy records, documented comorbidities, and laboratory testing was performed on the entire cohort (JS, BL, JM, FU). The elevation of residence was approximated using the average elevation computed from the US Census Zip Code Tabulation Areas Gazetteer File of the billing zip code [11]. Sleep testing reports were reviewed to classify the overall apnea-hypopnea index (AHI) and the proportion of central vs. obstructive events. Given the lack of inter-observer agreement when designating hypopneas as central or obstructive [12], all hypopneas were considered obstructive unless the study’s interpretation specifically stated that hypopneas were central. Central events (apneas and hypopneas) are referred to as central apneas for brevity. In addition to the 50% central apnea threshold implied by the ICSD-3 definition of CSA syndrome[1], we classified patients as < 10% central apneas or > 90% central apneas to separate pure obstructive and pure central sleep apnea, respectively.

The etiologies of central apneas were categorized by the presence of comorbidities known to cause CSA (cardiac, neurologic, opiate, treatment-emergent), which were presumed to be the cause of central apneas when present. A nonstandard designation, **C***entral***A***pneas occurring in predominantly***O***bstructive***S***leep***A***pnea* (CA-OSA) was utilized for patients where central apneas constituted less than 50% of total events but were not post-arousal or sleep-onset related in the polysomnogram report, were present before treatment, and were not associated with another known cause of CSA. Operational definitions used to classify the etiology of CSA are listed in Table 1 [3, 13–21]. Diagnostic tests of patients with primary CSA were reviewed (KW, KMS) to ensure the accuracy of diagnosis.

### Outcomes:

**Figure 1:**
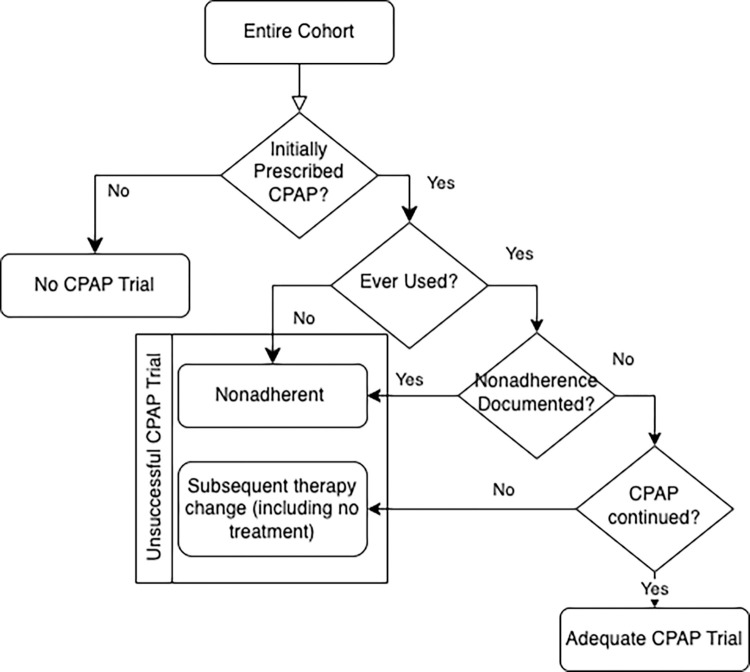
Outcome Classification Algorithm. Both fixed-pressure and auto-titrating CPAP were considered CPAP. The absence of usage or documented abandonment of therapy, as documented by the treating provider, was used to define adherence. Therapy change was determined by a review of sleep provider notes. We define “adequate” and “unsuccessful” trials of CPAP based on subsequent patient or provider behavior rather than physiologic normalization or clinical outcomes, which assumes that patients and providers will continue therapy when they benefit from it and switch or discontinue treatment that is not helping them.

Patients were categorized as having an initial trial of CPAP if the first prescription for sleep-disordered breathing was for CPAP (fixed pressure or auto-titrating). Patients initially treated with ASV, Bilevel Positive Airway Pressure (BPAP), other non-invasive ventilation (NIV) modes, supplemental oxygen, mandibular advancement device, or no treatment were categorized as having not received a trial of CPAP. Patients prescribed CPAP were classified as having an unsuccessful CPAP trial if they had documented nonadherence by their sleep provider or a subsequent change to a different modality (including no treatment). Patients who were adherent and continued CPAP were considered to have an adequate CPAP trial (Fig. 1). Follow-up was assessed in all ensuing notes (summarizing 6 to 60 months of clinical course after the initial sleep study).

### Statistical Analysis:

Logistic regression was used to model the likelihood of receiving an initial prescription for CPAP. Independent variables (age, smoking status, BMI, AHI, diagnostic testing, etiology, and ordinal category of percentage central apneas) were selected a priori based on subject knowledge [22]. For patients prescribed CPAP, a second logistic regression using the same predictors was used to model the likelihood of an adequate CPAP trial (no subsequent change from CPAP to another modality or nonadherence). Differences between predictors of an initial CPAP trial and subsequent course were assessed using unequal variance t-tests on the logit scale. Two sensitivity analyses, using inverse probability of treatment weighting and multinomial logistic regression, were performed to evaluate if differences between patients prescribed CPAP and those who were not would explain diverging response rates. Predictors of nonadherence were also modeled using logistic regression. The strength of associations are presented as odds ratios (OR) and average marginal effects, which estimate sample-averaged risk differences [23]. The missing data rate was below 5%, and thus no imputation was performed [24]. Statistical analyses were performed using Stata version 17 (StataCorp, College Station, TX).

## Results

**Figure 2:**
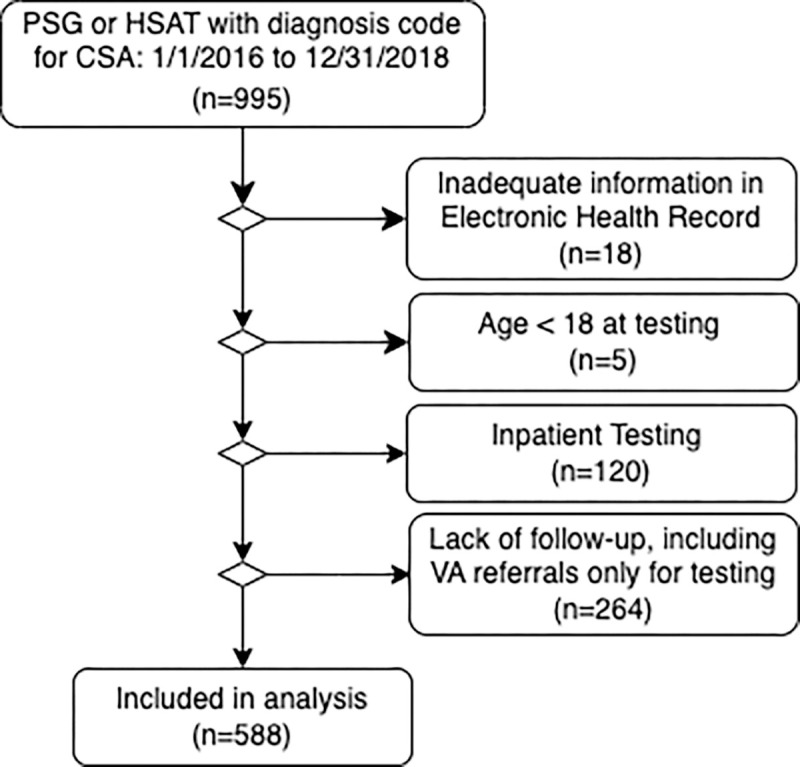
Enrollment Flow Diagram. PSG = polysomnography, HSAT = Type 3 home sleep apnea testing (Nox T3, Nox Medical USA, Suwanee, GA, USA), CSA = Central sleep apnea, VA = Salt Lake City George Whalen Veterans Affairs Hospital, a local hospital without a sleep laboratory and with a separate medical record system.

Out of 995 patients identified as having a diagnosis code for CSA between Jan 2016 and Dec 2018, 588 patients were included in the final analysis (reasons for exclusions are shown in Fig. 2). The median age in the cohort was 60 years (Interquartile Range [IQR] 47 to 70 years). Most were white or ‘not Hispanic/Latino’ (35% and 58.2%) and male (70%). Few smoked (8.3% current, 29% prior). Comorbidities were common (hypertension in 56%, diabetes in 22%, coronary artery disease in 18%, and a psychiatric or mood diagnosis in 39%). The median residence elevation was 4673 ft above sea level (IQR 4322, 5221 ft; Supplement, S1). A minority of patients were diagnosed with HSAT (28%, n = 162, Supplement, S2).

Most patients had moderate-to-severe sleep apnea (median AHI of 36 events/hour, IQR 20 to 62), and most patients had significant overlap with OSA. Despite receiving diagnostic codes for CSA, only 80 (14%) patients had more than 50% central apneas in their diagnostic sleep study, and only 1.9% (11 of 588) had over 90% central apneas. TECSA was the most common cause of central apneas (35%), followed by cardiac causes (27%, most commonly atrial fibrillation and heart failure with preserved ejection fraction), neurologic causes (19%, most commonly stroke), CA-OSA (14%), and opiates (11%). Primary CSA accounted for only 1.5% (9 of 588) of patients with central sleep apnea diagnoses.

**Figure 3:**
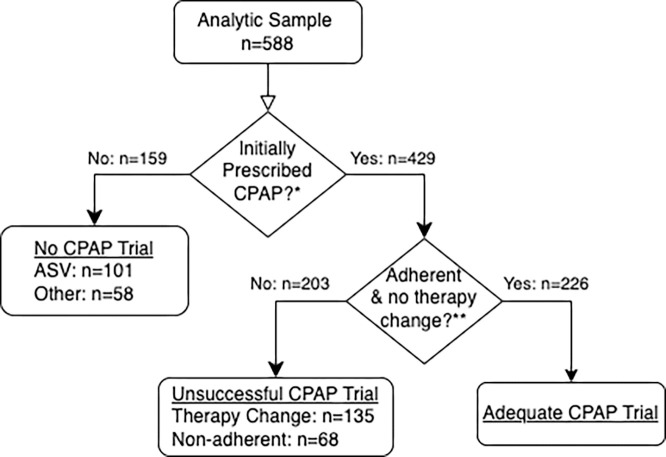
Outcomes Flow Diagram * Factors influencing the decision to prescribe CPAP initially are modeled in the first regression model, represented by dark grey/dashed in Figure 4 ** Factors influencing the likelihood of an adequate CPAP trial are modeled in the second logistic regression, represented by light grey/solid in Figure 4 Of the 135 that underwent a therapy change, 83 were changed to ASV. The remaining 52 were changed to either Bilevel Positive Airway Pressure (BPAP), supplemental oxygen, no treatment, or a different noninvasive ventilator modes.

Most patients received an initial trial of CPAP (73%, 429 of 588), including many with greater than 50% central apneas (49%, 39 of 80; Table 2). Seventeen percent (101 of 588) of patients proceeded directly to ASV, with the remaining receiving BPAP, oxygen, or no treatment (Fig. 3). Fifty-three percent (226 of 429) of all patients who received CPAP had an adequate CPAP trial (Table 3). Of the 47% who had unsuccessful trials of CPAP, 16% (68 of 429) were nonadherent to therapy, and 32% (135 of 429) were switched to alternative or no treatment (ASV in 83 of 135). In the subgroup of patients with more than 50% central apneas who received a CPAP trial, 31% (12 of 39) had adequate response to CPAP (Supplement, S3).

### Predictors of initial prescription of CPAP (Fig. 4):

In the multivariable model, a diagnosis made by HSAT (18% more likely [95% confidence interval 10%, 27%]) and a lower percentage of central apneas (< 10% central: 23% more likely [8%, 37%], 10–49.9% central 13% more likely [0%, 27%] as compared to 50–89.9% central apneas) independently predicted receiving an initial trial of CPAP. Conversely, older patients (4% less likely per decade [1%, 6%]), patients experiencing CSA due to opiates (21% less likely [6%, 35%]), and those that had > 90% central apneas (42% less likely [16%, 67%] as compared to 50–89.9% central) independently correlated with a lower likelihood of receiving a CPAP trial.

### Predictors of an adequate trial among those initially prescribed CPAP (Fig. 4):

After adjusting for other covariates, patients who had less than 50% central apneas were more likely to have an adequate response to the CPAP trial (< 10% central: 25% more likely [6%, 51%]; 10–49.9% central: 22% more likely [3%, 41%] as compared to 50–89.9% central apneas). Conversely, current smoking was predictive of an unsuccessful CPAP trial (31% less likely [14%, 48%]), likely because current smoking was strongly independently associated with risk for nonadherence (24% more likely to be nonadherent [5%, 43%]; Supplement S4). CSA associated with opiates also predicted an increased risk of unsuccessful CPAP trial (23% less likely to be adequate [3%, 49%]), but age, sex, BMI, severity of AHI elevation, and other etiologies of CSA were not associated likelihood of an unsuccessful CPAP trial.

### Concordance between the decision to trial CPAP and the likelihood of adequate response:

Older age (less likely to receive CPAP, but no difference in the likelihood of unsuccessful CPAP trial, P = 0.03) and CA-OSA (tended toward lower likelihood of receiving CPAP but more likely to have an adequate treatment response, P = 0.02) were directionally discordant. Diagnosis with HSAT and < 50% central apneas were both concordantly associated with an increased likelihood of CPAP trial and an increased chance of an adequate response when trialed with initial CPAP. Patients with CSA due to opiates were less likely to receive CPAP and also less likely to have an adequate response. Elevation of residence was not independently associated with either the likelihood of CPAP prescription, the likelihood of adequate treatment response to CPAP, or the odds of nonadherence. Sensitivity analyses showed similar findings (Supplement, S5)

## Discussion

Scant evidence has been reported on what features clinicians currently use, and should use, to predict which patients with central apneas will benefit from an initial trial of CPAP, as opposed to proceeding to another modality such as ASV. We report on several demographic, etiologic, and sleep study parameters that independently associated with the prescription of an initial CPAP trial, the likelihood of an adequate response, or both:

### Patient characteristics:

Prior work suggests older age is associated with ventilatory control instability [25–27], which may explain why providers in this study were less likely to prescribe CPAP in older adults. However, this physiologic rationale did not translate to a higher CPAP failure rate in practice, perhaps because CPAP sufficiently stabilizes ventilatory control[28] or pharyngeal collapsibility has an even greater contribution to pathogenesis in older adults[29]. The discordance between provider-predicted response (represented by the likelihood of initial prescription for CPAP) and the likelihood of an adequate outcome suggests prescribing patterns might be improved for this group of patients.

### Etiology:

This study corroborates previously described low response rates to CPAP for patients with opiate use [30] and higher CPAP response rates when central apneas occur in the setting of OSA, as in TECSA [31; 32]. Prior data suggest roughly half of the patients with CSA related to CHF have normalization of the AHI with CPAP [33], but we are unaware of previous comparative data between etiologies. We found that central apneas cause by cardiac or neurologic conditions respond to CPAP at roughly average covariate-adjusted rates. Similar to prior reports [18; 34], we found that primary CSA is very rare (9 of 588), and thus conclusions about CPAP prescribing or responsiveness remain tentative.

### The proportion of central vs. obstructive events:

Like prior studies, we find that most patients with central apneas have substantial overlap with OSA (only 11 of 588 had > 90% central apneas)[21; 35]. Current management is implicitly dichotomized at the threshold of 50% central events that defines whether a patient is labeled as principally OSA or CSA[1]. We find a “dose”-response relationship, where patients in a higher percentage of central events category were less likely to receive a CPAP trial and less likely to have an adequate response when trialed. This suggests that the proportion of central events might be better conceptualized as a continuous spectrum. Future work utilizing improved event-level differentiation between central and obstructive events [36–38] or endotyping patient-level features [39; 40], such as predisposition for upper-airway collapse and ventilation control abnormalities, have the potential to refine this paradigm further.

Relatedly, one role of a disease definition is to delineate patients expected to respond to specific treatments. However, for patients with opiate-related central apneas, rates of an adequate CPAP trial were low even when central apneas constituted < 50% of total events. Therefore, opiate-associated central apneas might be considered “central sleep apnea syndrome” even when the proportion of central apneas is lower than 50%.

### Care delivery parameters:

Patients diagnosed with HSAT had a higher rate of initial CPAP trial and low rates of subsequent nonadherence or modality switch, even after controlling for etiology and other factors (see Supplement, S2). This suggests that unexpected identification of central apneas on home testing is often successfully treated with CPAP. This is reassuring, given trends toward HSATs due to insurance requirements, the COVID pandemic, and the convenience of HSATs that have led to more frequent identification of CSA on HSAT in recent years.

Our center is at a comparatively high elevation for the United States (4500ft above sea level), which may influence the likelihood [13] and physiology [14] of CSA. However, we did not find evidence that the elevation of residence influenced provider prescribing or rates of adequate response to CPAP.

### Patterns of breathing:

Lastly, Cheyne-Stokes breathing [41], ataxic breathing [42], or other information contained in polysomnograms [40] may reveal aspects of physiology that predict CPAP responsiveness, but they are not currently utilized in guideline-directed management algorithms [5; 6; 8]. The presence of these patterns was not systematically documented, and thus we cannot evaluate their current usage or predictive value. Furthermore, if providers accurately use these features, the patients who received CPAP may differ from those who did not, even after matching and propensity weighting by observed characteristics [43].

In sum, we observed that clinicians often match their frequency of initial CPAP prescription for patients with central apneas to characteristics that independently predict an adequate response to CPAP. However, clinicians were less likely to prescribe an initial CPAP trial in older patients and patients with CA-OSA than their response rate to CPAP would suggest. These findings should help clinicians better individualize their initial treatment recommendations for patients with central apneas.

## Figures and Tables

**Figure 4:**
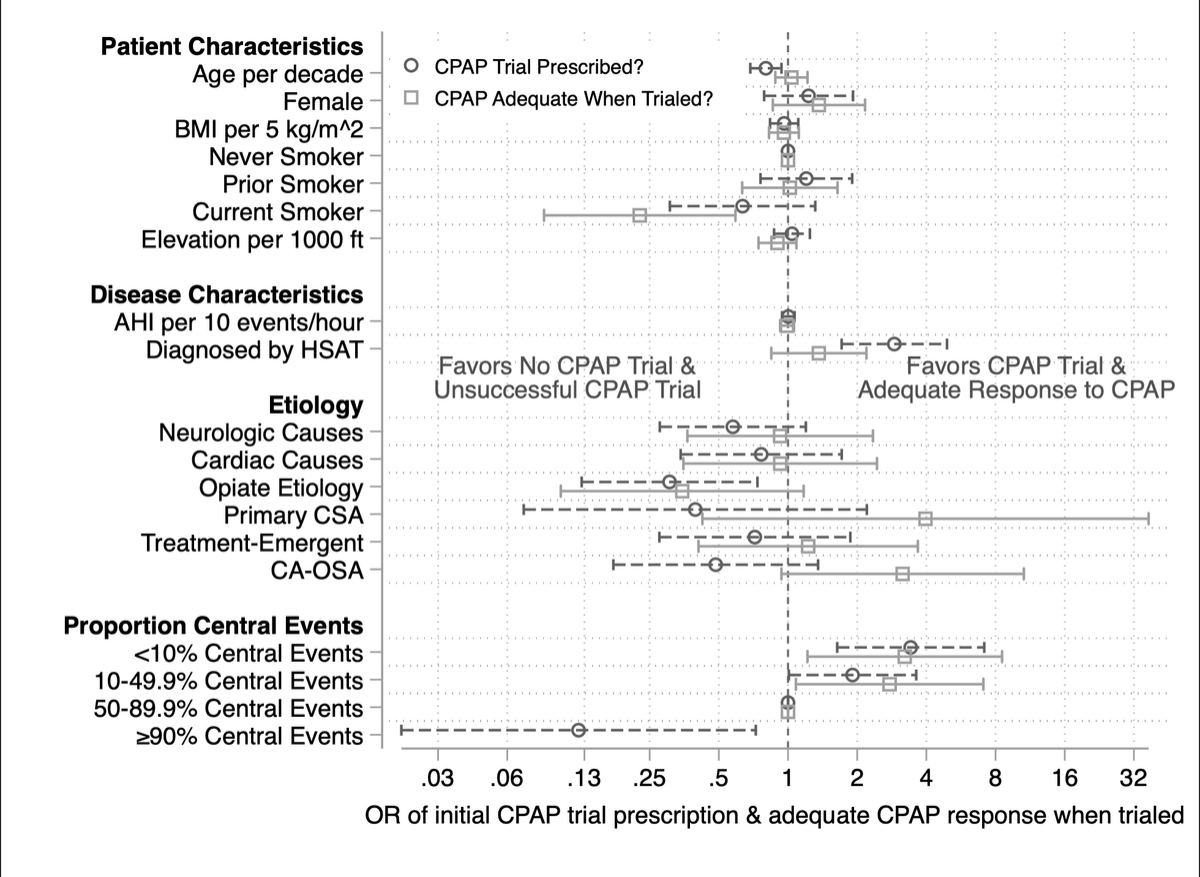
Factors associated with an initial prescription for CPAP and the likelihood of adequate response to CPAP. Multivariable logistic regression with the factors predicting the odds of a patient receiving an initial prescription for CPAP are represented in dark grey-dashed (circle). A second multivariable logistic regression using the same independent variables predicts the odds of an adequate CPAP trial, defined as no further adjustments to therapy and no documented nonadherence, shown in light grey-solid (square). Odds ratios (OR) and 95% confidence intervals are presented. Factors in which the odds ratios differ between the two models are ones. in which providers implied predictions about the likelihood of an adequate response to CPAP may be suboptimal. There were not enough patients with pure CSA (>90% central apneas) who were prescribed an initial trial of CPAP (n=2) to estimate the likelihood of a successful trial.

**Table 1 :** Etiology definitions

Etiology*	Operational Definition
Cardiac causes	Heart failure with reduced ejection fraction (HFrEF), heart failure with preserved ejection fraction (HFpEF), atrial fibrillation, or atrial flutter
Neurologic causes	Ischemic or hemorrhagic stroke, traumatic brain injury, neurodegenerative disorders, or Arnold-Chiari malformation.
Opiate-associated CSA	Specifically, methadone, fentanyl, oxycodone slow-release, buprenorphine, or shortacting opiates with a dosage of more than 200 morphine equivalents daily. Baclofen was also included in this category.
Treatment-emergent CSA (TECSA)	Patients were categorized as having TECSA if they had no other known causes of central apneas, did not have central apneas present on the diagnostic portion of the sleep study but developed central apneas in response to continuous positive airway pressure (CPAP) therapy. Patients who only developed central apneas in the context of treatment but had cardiac, neurologic, or opiate-use comorbidities were classified according to comorbidity rather than TECSA.
Central apneas occurring in the context of predominantly OSA (CA-OSA)	Patients were given the nonstandard designation *‘CA-OSA’* if they had central apneas that constituted the minority of events (<50%) and occurred in the context of obstructive sleep apnea, occurred in the absence of other known causes (cardiac diseases, neurologic disease, or medications), were not post-arousal or sleep-onset related in the sleep testing report, and were present before any treatment. In prior reports, these patients have been termed “treatment persistent CSA” if central apneas continued after PAP treatment [31].
Primary CSA	Patients with >50% central apnea without other known causes of central apnea were labeled as primary CSA.

* If a patient had multiple causative conditions (Cardiac, Neurologic, Opiate), such as both a history of stroke and atrial fibrillation, patients were included in both groups.

**Table 2:** Sample characteristics, stratified by whether patients received a trial of CPAP

	All	Received Initial CPAP Prescription	No Initial CPAP Trial	P-value
	N=588	N=429	N=159	
**Patient Characteristics**				
Age (years)	60 (47–70)	58 (47–70)	62 (51–71)	0.032
Female	30% (177)	31% (132)	28% (45)	0.56
BMI (kg/m^2^)	31.1 (27.6–36.3)	31.4 (28.0–36.3)	30.8 (26.3–36.7)	0.28
Smoking Status				0.28
Never Smoker	63% (370)	64% (274)	60% (96)	
Prior Smoker	29% (169)	29% (124)	28% (45)	
Current Smoker	8% (49)	7% (31)	11% (18)	
Elevation (feet), home zip-code	4672.5 (4322.0–5221.0)	4685.00 (4322.0–5221.0)	4486.0 (4275.0–4943.0)	0.076
**Disease Characteristics**				
AHI (events/hour)	36 (20–62)	35 (20–59)	40 (22–73)	0.12
Diagnosed by HSAT	28% (162)	32% (136)	16% (26)	<0.001
Cardiac Cause	27% (158)	26% (110)	30% (48)	0.27
Neurologic Cause	19% (114)	18% (77)	23% (37)	0.15
Opiate Cause	11% (67)	8% (34)	21% (33)	<0.001
Primary CSA	1.5% (9)	1% (5)	3% (4)	0.24
TECSA	35% (207)	39% (169)	24% (38)	<0.001
CA-OSA	14% (82)	14% (62)	13% (20)	0.56
Percentage of Central Apneas				<0.001
<10% Central Apneas	30% (175)	34% (144)	19% (31)	
10–49.9% Central Apneas	57% (333)	57% (246)	55% (87)	
50–89.9% Central Apneas	12% (69)	9% (37)	20% (32)	
90+% Central Apneas	2% (11)	0% (2)	6% (9)	

**Table 3:** Characteristics of patients who were prescribed an initial CPAP trial, stratified by outcome

	Adequate CPAP Response	Unsuccessful CPAP trial	P-value
	N=226 of 429	N=203 of 429	
**Patient Characteristics**			
Age (years)	59.5 (47–70)	57 (47–70)	0.64
Female	34% (76)	28% (56)	0.18
BMI (kg/m^2^)	31.4 (28.0–35.7)	31.4 (27.9–37.0)	0.78
Smoking Status			0.006
Never Smoker	68% (154)	59% (120)	
Prior Smoker	28% (64)	30% (60)	
Current Smoker	4% (8)	11% (23)	
Elevation (feet), home zip-code	4718.0 (4402.0–5221.0)	4580.0 (4291.0–5221.0)	0.17
**Disease Characteristics**			
AHI (events/hour)	34 (18–57)	35 (21–62)	0.38
Diagnosed by HSAT	36% (81)	27% (55)	0.052
Cardiac Cause	23% (52)	29% (58)	0.19
Neurologic Cause	15% (35)	21% (42)	0.16
Opiate Cause	3% (7)	13% (27)	<0.001
Primary CSA	1% (3)	1% (2)	0.74
TECSA	42% (94)	37% (75)	0.33
CA-OSA	21% (47)	7% (15)	<0.001
Percentage of Central Apneas			0.006
<10% Central Apneas	35% (79)	32% (65)	
10–49.9% Central Apneas	60% (135)	55% (111)	
50–89.9% Central Apneas	4% (10)	13% (27)	
90+% Central Apneas	1% (2)	0% (0)	

## Abbreviations

CSA: Central sleep apnea
OSA: Obstructive sleep apnea
ICSD: International Classification of Sleep Disorders
ICD: International Classification of Diseases
IQR: interquartile range
CSR: Cheyne-Stokes respiration
CPAP: Continuous positive airway pressure
ASV: Adaptive servo-ventilation
BPAP: Bilevel positive airway pressure
TECSA: Treatment-emergent central sleep apnea
PSG: Polysomnography
HSAT: Home sleep apnea test
AHI: Apnea-hypopnea index
BMI: Body mass index
HFrEF: Heart failure with reduced ejection fraction
HFpEF: Heart failure with preserved ejection fraction
CA-OSA: Apneas that occur in the context of predominantly obstructive sleep apnea, without alternative cause (present before CPAP any treatment, are not associated with arousal or sleep-onset, and happen in the absence of other known causes of CSA)
OR: odds ratio
